# Human microglia reduce alpha-synuclein aggregation and are neuroprotective in adult mouse brain

**DOI:** 10.1016/j.bbi.2025.106097

**Published:** 2025-09-03

**Authors:** Katrina Albert, Sanni Peltonen, Anni Vanne, Sara Kälvälä, Valtteri Syvänen, Jari Koistinaho, Kelvin C. Luk, Šárka Lehtonen

**Affiliations:** a A.I. Virtanen Institute for Molecular Sciences, University of Eastern Finland, 70211 Kuopio, Finland; b Helsinki Institute of Life Science, University of Helsinki 00014 Helsinki, Finland; c Drug Research Program, Division of Pharmacology and Pharmacotherapy, University of Helsinki 00014 Helsinki, Finland; d Center for Neurodegenerative Disease Research, Department of Pathology and Laboratory Medicine, University of Pennsylvania, Perelman School of Medicine, Philadelphia, PA 19104-4238, USA

**Keywords:** Microglia, Xenotransplant, Parkinson’s disease, Alpha-synuclein, Hypocretin, RNAseq, Preformed fibrils

## Abstract

Microglia, brain-resident immune cells, are involved in pathophysiology of several neurodegenerative diseases, including Parkinson’s disease. Given significant species-specific differences in microglia gene expression, particularly in disease-risk genes, as well as the highly reactive nature of these cells, studying human microglia in a whole brain environment is essential. Here, we established a humanized mouse model by transplanting human induced pluripotent stem cell-derived hematopoietic progenitor cells into the striatum of immunodeficient adult mice and injected human alpha-synuclein preformed fibrils to model Parkinson’s disease pathology. Transplanted human cells engraft, mature into microglia and maintain their phenotype for at least three months post-transplantation. These human microglia interact with alpha-synuclein, significantly limiting its propagation from the striatum to the substantia nigra and further reducing local small aggregates; they also mildly protect tyrosine hydroxylase neurons there. Transcriptomic profiling reveals 56 differentially expressed genes in human microglia in response to alpha-synuclein preformed fibrils, while host mouse cells show 202 gene expression changes, including an upregulation of gene Hcrt (fold change = 7.77, p = 0.0015). Immunohistochemistry analysis further confirms the preservation of hypocretin-positive neurons in the hypothalamus of the transplanted mice (p = 0.0079). The findings highlight the neuroprotective role of human microglia and establish a more disease-relevant *in vivo* model for investigating alpha-synuclein aggregation and therapeutic interventions in Parkinson’s disease.

## 1. Introduction

As the macrophages of the CNS, microglia act as immune cells where in adult brain they survey the surrounding environment and react to insults, release soluble factors, and phagocytose debris. Researchers have recently gained insights from studying human microglia and observe key differences in their gene expression compared to mouse microglia, including expression of neurodegenerative disease-related genes (Gosselin et al., 2017). Utilizing microglia derived from human induced pluripotent stem cells (iPSCs) therefore offers an ethical and relevant way to dissect mechanism of human brain diseases. A caveat to this is that human iPSC-derived microglia as well as human microglia *in vitro* show divergent gene expression compared to *ex vivo* human microglia – and microglia transplanted to the neonatal mouse brain (Hasselmann et al., 2019). Human hematopoietic progenitor cells (HPCs) derived from embryonic stem cells (Mancuso et al., 2019) and iPSCs (Hasselmann et al., 2019; Xu et al., 2020) are able to survive, proliferate, and mature to microglia with a brain-resident phenotype when transplanted to neonatal immunodeficient mice. These immunodeficient mice have human macrophage colony stimulating factor (M-CSF) knocked in to specifically allow for transplant of human hematopoietic cells such as microglia (Rathinam et al., 2011). Examination of these xenotransplant microglia in Alzheimer’s disease models show a human-specific response to disease pathology at the transcriptomic level that differs from mouse, clearly demonstrating the necessity of this context (Hasselmann et al., 2019; Mancuso et al., 2019; Mancuso et al., 2024).

Microglia are also relevant in neurodegenerative disorder Parkinson’s disease (Smajić et al., 2022) where increasing evidence shows neuroinflammation plays a role in its pathophysiology (Tansey et al., 2022). The main hallmarks of Parkinson’s disease are loss of dopamine neurons of the substantia nigra pars compacta along with neuronal accumulation of small protein α-synuclein into Lewy bodies and neurites. We and others show that human iPSC-derived microglia uptake fibrillar α-synuclein (Niskanen et al., 2024; Scheiblich et al., 2021; Rostami et al., 2021) *in vitro*, and are able to mitigate toxic aggregation from nearby neurons (Scheiblich et al., 2024). Conversely, aggregated α-synuclein causes a proinflammatory activation state in human iPSC-derived microglia (Trudler et al., 2021; Yildirim-Balatan et al., 2024), which may negatively affect ability of the cells to phagocytose aberrant proteins (Niskanen et al., 2024). Maintaining microglia functionality to aid in fully operational CNS homeostasis is therefore an attractive therapeutic strategy in Parkinson’s disease and other proteinopathies.

Modelling the hallmarks of Parkinson’s *in vivo* can be accomplished by injection of α-synuclein preformed fibrils (PFFs) to the CNS where they recruit endogenous α-synuclein to form aggregates that spread to connected brain areas (Luk et al., 2012). These aggregates are positive for α-synuclein phosphorylated at serine 129 (pS129) and over time recapitulate patient Lewy pathology (Mahul-Mellier et al., 2020). α-synuclein PFFs injected to rodent brain is a useful tool to understand how α-synuclein aggregation may affect neuronal health (Patterson et al., 2024) and in testing of novel therapies (Albert et al., 2021); the aforementioned human iPSC-derived microglia studies have utilized these fibrils as well. However, the latter inherently lacks more complex interaction with other cells of the brain, while the former uses mouse cells only, not accounting for species variation. Therefore, we aimed to create a novel Parkinson’s disease model where we transplanted human iPSC-derived human microglia progenitors to immunodeficient mice and injected them with α-synuclein PFFs.

## 2. Materials and methods

### 2.1. Mice

Human M-CSF knockin mice (C;129S4-*Rag2*^*tm1.1Flv*^*Csf1*^*tm1(CSF1) Flv*^*Il2rg*^*tm1.1Flv*^/J) were obtained from The Jackson Laboratory (strain #017708). Both male and female mice were used and were 3 months old at the start of the experiments (*N* = 60). We are the first to use adult mice in this context as we aimed to develop a more disease-relevant model. All experiments were carried out at the University of Eastern Finland Laboratory Animal Centre. Mice were housed in groups under a 12 h light/dark cycle with *ad libitum* access to food and water in a room with constant temperature and humidity; environmental enrichment included pipes and bedding materials. All animal experiments were approved by the Finnish National Board of Animal Experiments and were carried out according to the European Community guidelines for the use of experimental animals under license numbers ESAVI/2938/2021, ESAVI/12318/2024. All guidelines for reporting the use of the animals were followed and 3R principles were adhered to. Mice were randomly assigned to treatment groups.

### 2.2. Hemopoietic progenitor cell differentiation from human induced pluripotent stem cells

Human iPSCs were 2 lines from a female (previously generated and characterized (Holmqvist et al., 2016) and a male healthy donor (commercial line, Takara Bio, Y00325). Approval for the use of patient-derived material from Hospital District of Northern Savo Research Ethics Committee (123/13.02.00/2016).

HPCs were differentiated as previously (Niskanen et al., 2024) from a modified protocol from (Abud et al., 2017; McQuade et al., 2018). Briefly, the human iPSCs were detached as colonies with ReLeSR and plated on Matrigel coated 6-well plates in E8-media. The STEMdiff hematopoietic Kit (StemCell Technologies) was used for the differentiation of the HPCs. The day after plating the differentiation was started (day 0 of the differentiation). The cells were maintained in STEMdiff hematopoietic basal medium with supplement A for 3 days and then in STEMdiff hematopoietic basal medium with supplement B for the rest of the cultures. The day before cell collection, the wells were cleaned to remove dead cells from the media. The detached HPCs were collected from the media between days 10 and 15 of the differentiations. The HPCs were further differentiated to microglia (as in) (Niskanen et al., 2024) to ensure the best chance of differentiation *in vivo*. Herein, we refer to HPCs as microglia progenitors when transplanted.

### 2.3. Preparation of alpha-synuclein preformed fibrils

Human α-synuclein PFFs were provided by Kelvin C. Luk (University of Pennsylvania) and were prepared as described previously (Luk et al., 2012). Monomeric human α-synuclein expressed in *E. coli* was purified, thawed, then centrifuged to pellet the aggregated material, the supernatant was then removed and diluted in a separate tube. This tube then underwent shaking at 1000 rpm for 7 days at +37 °C to ensure formation of fibrillar structures. Aliquots were stored at 80 °C until dilution. On the day of surgeries, the PFFs were thawed at RT and diluted to 2.5 mg/mL in sterile PBS under the fume hood. They were then sonicated in a cooled sonicator bath (Bioruptor, Diagenode) for 10 cycles with 30 s on and 30 s off. All surfaces were wiped with 1 % SDS and used tips put into a separate sealed container containing 1 % SDS.

### 2.4. Stereotaxic surgeries

All stereotaxic surgeries were performed with the mice under isoflurane anesthesia (3.5 % induction, 1.5–2 % maintenance). The mice were placed in a stereotaxic frame (Stoelting) and ~0.1 mL of lidocaine was given subcutaneously to the skin on top of the head to anesthetize the area and stem the bleeding, then a small incision was made to expose the skull. Burr holes were then made with a micro drill. 33G steel needle with a 10 μL syringe (Nanofil, World Precision Instruments) was used in all experiments. All mice received subcutaneous injection of carprofen for pain relief (Rimadyl, Pfizer, 20 mg/kg). Mice were placed in a separate warmed recovery box before being returned to their home cage.

#### 2.4.1. Human hemopoietic progenitor cell transplant

The human HPCs were donor sex-matched to the mouse sex used for transplantation. Cells were collected into sterile saline just prior to surgery at a concentration of 50 000 cells/μl. Microglia progenitors were transplanted into 3 sites unilaterally in the striatum (coordinates from bregma: A/P + 0.7, M/L −2.2, D/V −3.0; A/P + 1.2, M/L −1.5, D/V −3.0; A/P + 1.0, M/L −1.3, D/V −4.0), volume 2 μl per site, and flow rate 0.25 μl/min. The needle was rested for 5 min after each injection to prevent backflow. After each injection, the needle and syringe were cleaned with 70 % ethanol and sterile water. For sham operated mice, the needle was inserted into the same 3 sites for the duration of an expected injection then removed.

As these mice are poor breeders, we did not obtain a high enough n for each treatment group to justify separating by sex and in checking the data there were no significant differences when doing so, however n was low or 0 for some groups.

#### 2.4.2. Alpha-synuclein preformed fibril injections

Before each injection, the tube with the diluted human α-synuclein PFFs (2.5 mg/mL) was lightly tapped to avoid forming clumps. 2.5 μl of PFFs (or vehicle) were injected either unilaterally or bilaterally to the striatum (coordinates from bregma: A/P + 1.0; M/L ± 1.5; D/V −3.5), flow rate 0.1 μl/min, and needle rested for 5 min. A separate needle was used for vehicle injection and the needles were cleaned in between injections with 1 % SDS.

### 2.5. Tissue collection for immunohistochemistry

For collection of the brains for immunohistochemistry, the mice were anesthetized with a near lethal dose of sodium pentobarbital (Euthoxin) given intraperitoneally and perfused transcardially with PBS then with 4 % PFA in PBS. The brains were removed immediately and put to 4 % PFA overnight at +4 °C, then to 20 % sucrose. Brains were then frozen in a cryostat (Leica CM3050 S), cut in 30 μm sections into PBS, immunostained immediately or put to a cryopreservant solution (20 % glycerol, 2 % DMSO in PBS) at −20 °C while awaiting further processing.

### 2.6. Immunohistochemistry

For immunofluorescent staining, free-floating sections were rinsed 3× with PBS, then put to blocking solution (4 % bovine serum albumin (BSA), 0.3 % Triton-X-100 in PBS) for 1 h, then incubated in the primary antibody in blocking solution (Table 1) overnight at +4 °C. The next day, the sections were rinsed with PBS 3× and then put to the secondary antibody (AlexaFluor goat anti-mouse, anti-rabbit, or anti-rat 488 or 568, 1:500) in blocking solution for 1–2 h. Sections were then rinsed again 3× with PBS and incubated with DAPI (1:2000 in PBS) for 15 min. Slides were rinsed with MilliQ water and put on to glass slides, air dried overnight, then coverslipped with Vectashield Mounting Media.

For the MJFR-14-6-4-2 antibody, antigen retrieval with 10 mM citrate buffer at +80 °C for 30 min was used.

#### 2.6.1. 3,3′-Diaminobenzidine (DAB) immunohistochemistry

For chromogenic DAB immunostaining, free-floating sections were rinsed 3× with PBS and endogenous peroxidase was blocked using 0.3 % of H_2_O_2_ in PBS for 30 min at RT. Sections were rinsed again 3× in PBS then blocked for 1 h in 4 % BSA, 0.3 % Triton-X-100 in PBS. The sections were then put to the primary antibody in blocking solution overnight at +4 °C. Next day, sections were rinsed with PBS 3× and put to the secondary antibody (Vector biotinylated secondary antibody goat antimouse (BA-2000) or anti-rabbit (BA-1000), 1:200) in blocking solution. Sections were rinsed 3× again with PBS, then incubated using the VECTASTAIN^®^ Elite ABC-HRP Kit, Peroxidase (Standard) (Vector, VEC-PK-6100) for 1 h, prepared according to the manufacturer’s instructions (1 drop of A per 5 ml + 1 drop of B per 5 ml, incubated 30 mins before use). Sections were rinsed in PBS 3× then developed using DAB tablets (D5905, Sigma) according to the manufacturer’s instructions. The sections were incubated in the DAB solution from approximately 45 s to 1 min and 30 s then immediately quenched in fresh PBS. Sections were rinsed 3× in PBS then put on to glass slides, and air dried overnight at RT. The next day the slides were dehydrated, coverslipped using Depex mounting media, and dried overnight before imaging.

### 2.7. Counting pS129 alpha-synuclein inclusions

All slides used for analysis were scanned for brightfield with a 20× objective on a VS200 slide scanner (Olympus). Olympus OlyVIA (Evident Technology) was used to view and image the immunostained sections. The number of pS129 α-synuclein positive inclusions were counted as in (Albert et al., 2021). An experimenter blind to the treatments manually annotated the immunostained inclusions in the substantia nigra area of each section (3 sections per mouse from approximately A/P − 4.5 to 6.0 relative to bregma) on each side. The results were calculated as average number of inclusions per mouse.

### 2.8. Alpha-synuclein aggregate pathology scoring

Semi-quantitative pathology scoring was performed as in (Chatterjee et al., 2019). The scoring was done by an experimenter blind to the treatments and corresponds to 0 = no aggregates, 1 = sparse aggregates, 2 = mild aggregates, 3 = dense aggregates, 4 = very dense aggregates. Aggregates were defined as positive signal from the MJFR-14-6-4-2 antibody in the substantia nigra area of each mouse.

### 2.9. Analysis of tyrosine hydroxylase neurons in the substantia nigra pars compacta

Slides were scanned and viewed as above. For comparative analysis of number of tyrosine hydroxylase (TH) neurons between each side of the substantia nigra pars compacta, MATLAB R2023b was used to count the number of cells as in (Penttinen et al., 2016). This method has shown a significant correlation (Pearson correlation *R* = 0.925, *P* < 0.001) when compared to using StereoInvestigator and the optical fractionator to count TH-positive cells, therefore it was used here to analyse the gradation of cell loss between the sides of the substantia nigra pars compacta. Images were taken from each substantia nigra area at 4× magnification (5–6 sections per mouse) and the pars compacta area was traced for each side. Using the MATLAB algorithm (available at) (Penttinen et al., 2016), each image was loaded to MATLAB and the number of cells were counted using parameters for cell size 30 and contrast to 195 (in some cases the contrast was changed to between 180 and 190 to account for differing background between mouse brains). This was performed by an experimenter blinded to the treatments. The number of cells were then averaged together for each side and presented as number of cells per side for each mouse.

### 2.10. Counting hypocretin neurons in the hypothalamus

Serial sections from the hypothalamus area of 5 mice with a total of 24 sections were immunostained for hypocretin-1/orexin A, scanned, and images taken as above. Positive cells were manually counted using the Fiji Cell-Counter plug-in. Results are represented as number of cells per section in the human microglia transplanted (hMG) vs. non-transplanted side (NT).

### 2.11. Collection and processing of samples for bulk RNA sequencing

For collection of samples for bulk RNA sequencing (RNAseq), 12 mice were deeply anesthetized and perfused as above with only cold PBS, then brains were collected and immediately flash frozen. The brains were then dissected while still frozen using a cryostat where the whole striatum from each side was collected using a brain hole punch, resulting in 24 samples. The frozen samples were shipped on dry ice to Genewiz (Leipzig, Germany). Total RNA was extracted using Qiagen RNeasy Plus Universal micro kit following the manufacturer’s instructions (Qiagen). RNA samples were quantified using Qubit 4.0 Fluorometer (Life Technologies) and RNA integrity was checked with RNA Kit on Agilent 5600 Fragment Analyzer (Agilent Technologies). ERCC RNA Spike-In Mix (#4456740, ThermoFisher Scientific), was added to normalized total RNA prior to library preparation following the manufacturer’s protocol. RNA sequencing libraries were prepared using the NEBNext Ultra II RNA Library Prep Kit for Illumina following the manufacturer’s instructions (NEB). Briefly, mRNAs were first enriched with Oligo(dT) beads. Enriched mRNAs were fragmented for 15 min at 94 °C. First strand and second strand cDNAs were subsequently synthesized. cDNA fragments were end repaired and adenylated at 3′ends, and universal adapters were ligated to cDNA fragments, followed by index addition and library enrichment by limited-cycle PCR. Sequencing libraries were validated using NGS Kit on the Agilent 5300 Fragment Analyzer and quantified by using the ubit 4.0 Fluorometer. The libraries were multiplexed and loaded on the flowcell on an Illumina NovaSeq X+ instrument according to the manufacturer’s instructions. The samples were sequenced using a 2 × 150 Pair-End (PE) configuration. Raw sequence data (.bcl files) generated from Illumina NovaSeq were converted into fastq files and de-multiplexed using Illumina bcl2fastq program version 2.20. One mismatch was allowed for index sequence identification.

### 2.12. Bulk RNA sequencing analysis

Reads in FASTQ format were sorted to human and mouse origin using BBsplit (Bushnell, n.d.) tool and reference genomes GRCh38 and GRCm39, respectively. It was shown that this tool can distinguish between human and mouse genes with up to 98 % accuracy and we have utilized this in a previous study with transplantation of human iPSC-derived astrocytes to the immunodeficient mouse and found that only 0.03 % of sequences misaligned between species (Koskuvi et al., 2022). Transcripts were quantified using Salmon (Patro et al., 2017). Differential gene expression was performed using the DEseq2 package (Love et al., 2014) and further analysis (i.e. significantly fold changed genes) using the edgeR package (Chen et al., 2025). Pathway analysis using ShinyGO 0.82 (Ge et al., 2020).

### 2.13. Statistical analysis

All statistical analyses were performed using GraphPad Prism 10 or R programming language in RStudio. All data represented as mean ± SD. Unpaired two tailed *t*-test or one- and two-way ANOVA with Tukey’s multiple comparison test for post hoc testing were used. For fold-change genes, P value cut-off of <0.05 or <0.01 were used. One outlier was removed from human microglia gene counts using Grubbs’ test with alpha = 0.05. Brown-Forsythe test was used to check that standard deviations were not significantly different (*P* < 0.05) when performing statistical testing.

### 2.14. Data availability

The data generated from bulk RNA sequencing is available at GEO, accession number: GSE298706.

## 3. Results

### 3.1. Human microglia progenitor cells transplanted to the adult mouse brain survive and express mature microglia markers

**Fig. 1.**
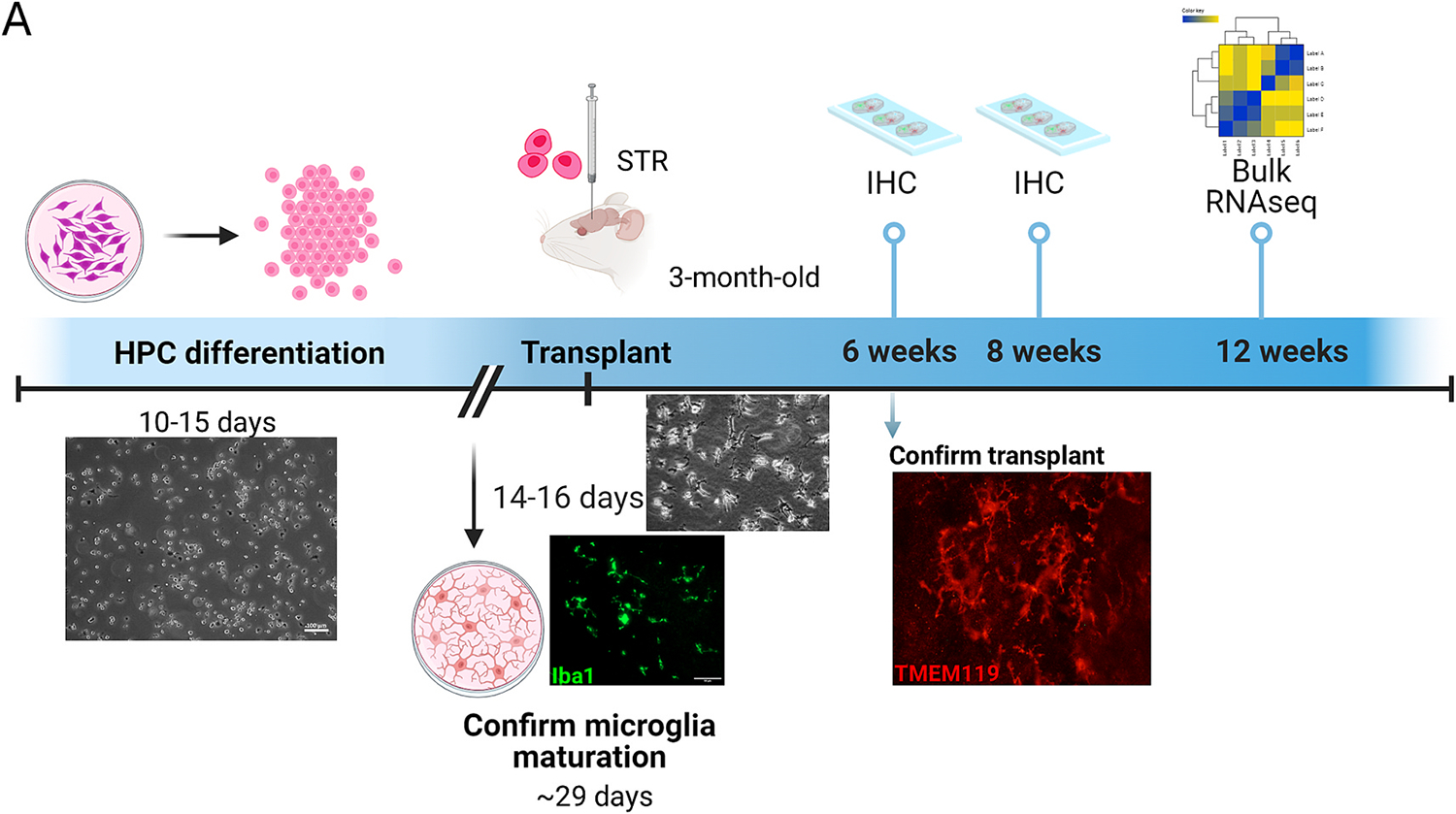
Derivation, transplantation, and outcome measures timeline. (A) Experimental timeline of transplantation of human hematopoietic progenitor cells (HPCs) to the striatum (STR) of 3-month-old human M-CSF knock-in mice. Human iPSC-derived HPCs transplanted at approximately 10–15 days. A portion of the cells were set aside and differentiated to microglia *in vitro* to confirm maturation (brightfield and Iba1 immunocytochemistry, scale bar 50 μm). At 6 weeks post-transplant, the presence of human TMEM119-positive cells in the striatum was confirmed by immunohistochemistry (IHC). IHC at 8 and 12 weeks and bulk RNA sequencing (RNAseq) at 12-weeks post-transplant.

**Fig. 2.**
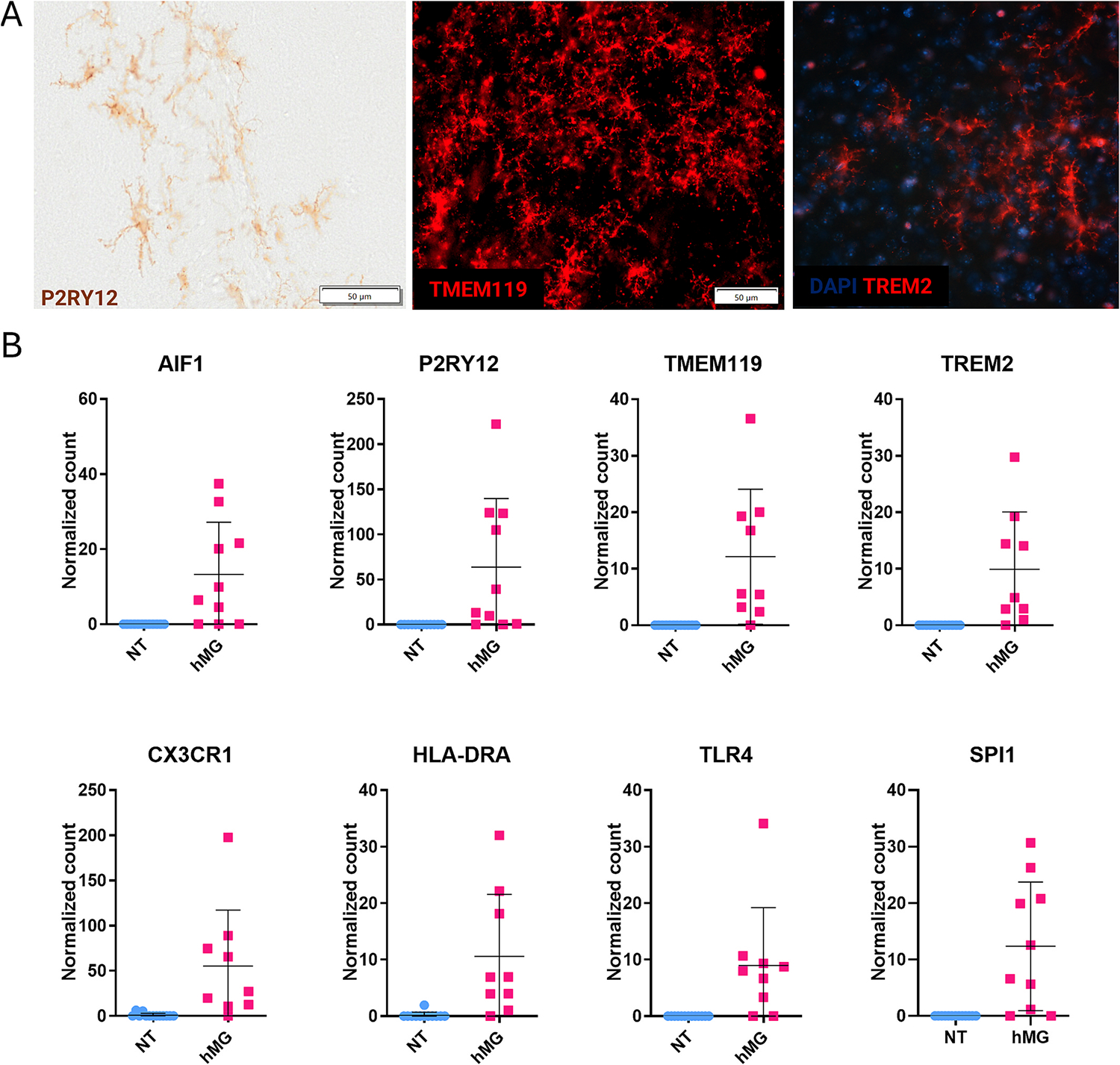
Human microglia progenitors survive and mature in adult mouse brain. (A) IHC from the striatum of transplanted mice demonstrating markers for human P2RY12, human TMEM119, and TREM2. Scale bar 50 μm. (B) Human normalized gene counts from bulk RNAseq of typical microglia markers comparing non-transplanted (NT) with transplanted (hMG).

We generated human HPCs from iPSCs and transplanted them at approximately 10–15 days differentiation to the striatum of 3-month-old (adult) human M-CSF knockin mice (Fig. 1A). We further cultured them to confirm development of a mature microglia phenotype *in vitro* (Iba1 immunocytochemistry) and at 6 weeks post-transplant confirmed the presence of ramified microglia cells positive for human transmembrane protein 119 (TMEM119) using immunohistochemistry. At 12 weeks post-transplant, the cells were positive for purinergic receptor P2Y12 (P2RY12), TMEM119, and triggering receptor expressed on myeloid cells 2 (TREM2) at the protein level (Fig. 2A), indicating the presence of mature human microglia. The transplanted human microglia (hMG) were checked with RNAseq and we confirmed expression of several human genes relevant for microglia: *AIF1*, *P2RY12*, *TMEM119*, *TREM2*, *CX3CR1*, *HLA-DRA*, *TLR4*, and *SPI1* (Fig. 2B).

### 3.2. Human microglia impede the retrograde propagation of alpha-synuclein aggregates in vivo

**Fig. 3.**
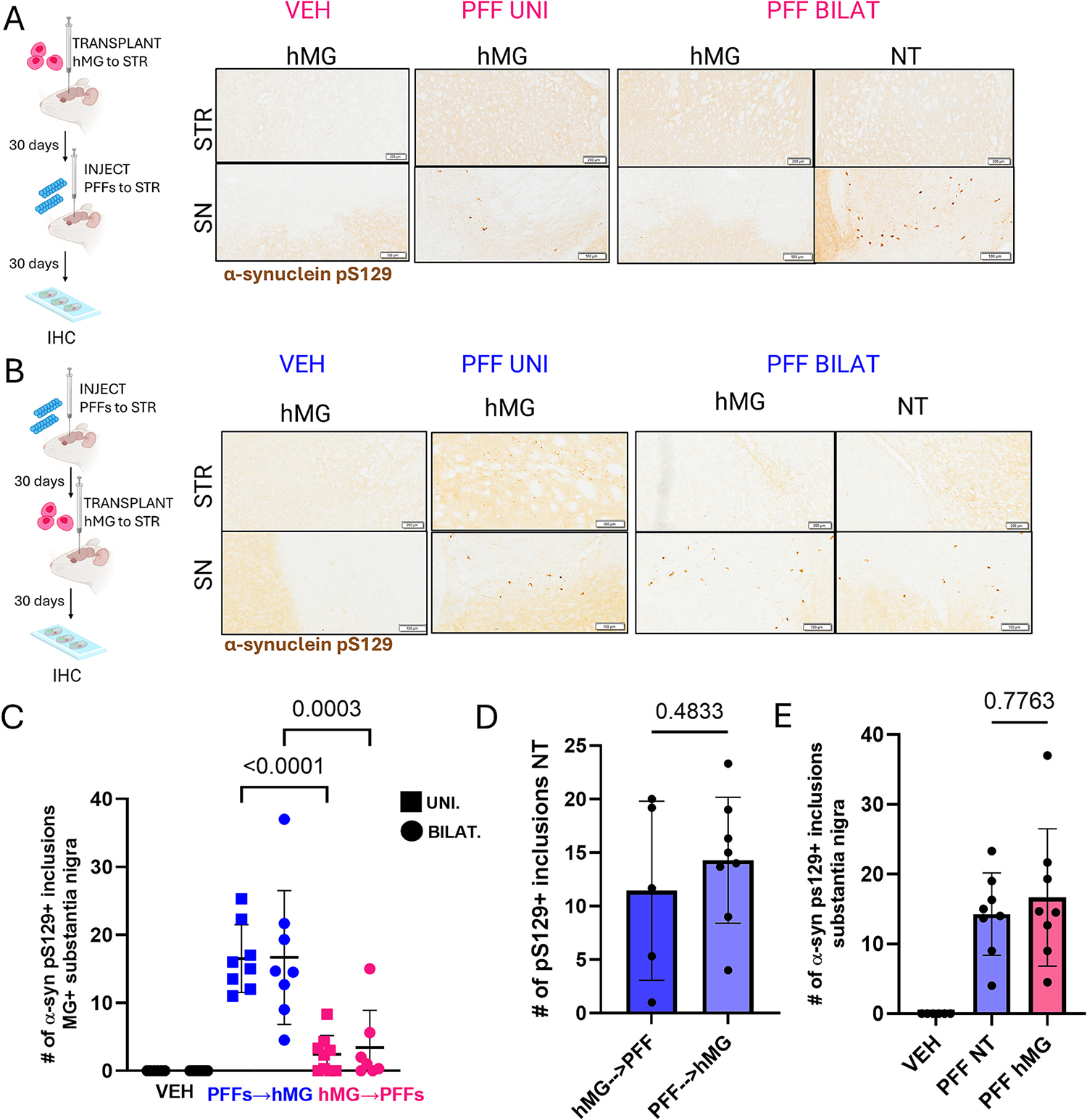
Alpha-synuclein aggregate spreading is affected by human microglia. (A) Transplantation of human microglia progenitors to the striatum (STR) 30 days prior to injection of α-synuclein PFFs to STR. α-synuclein phosphorylated at S129 (pS129) immunohistochemistry of vehicle (VEH)-treated, PFFs injected unilaterally to the same side as the transplantation (PFF UNI), and PFFs injected bilaterally (PFF BILAT) showing hMG (transplanted) side and NT (non-transplanted) side from both STR and substantia nigra (SN) 30 days after injections. (B) PFFs injected first to the STR, then 30 days later the human microglia progenitors were transplanted to the STR. Immunohistochemical images as in A. Scale bars STR 200 μm, SN 100 μm. (C) Quantification of the number of α-synuclein pS129-positive inclusions in the substantia nigra of the transplanted (hMG) side for all treatments in both paradigms. Mean ± SD. (D) Number of alpha-synuclein inclusions phosphorylated at S129 (pS129) in the non-transplanted side (NT) for both paradigms where human microglia progenitors are transplanted before PFF injection (hMG → PFF) compared to when they are transplanted after PFF injection (PFF → hMG). (E) Number of alpha-synuclein inclusions phosphorylated at S129 (pS129) in bilateral injection groups VEH and PFF in PFF → hMG paradigm.

Our previous *in vitro* data using human microglia and α-synuclein PFFs indicates that the cells take up the PFFs resulting in further downstream effects (Niskanen et al., 2024). We therefore tested a similar setup *in vivo* where human microglia progenitors were transplanted first to the striatum of human M-CSF knockin mice and then 30 days later human α-synuclein PFFs (or vehicle) were injected adjacently to the transplanted cells either unilaterally on the same side as the transplant or bilaterally. 30 days after PFF injections, brains were collected and α-synuclein phosphorylated at S129 (α-synuclein pS129) immunohistochemistry was performed on the striatum and substantia nigra of each mouse (Fig. 3A). No staining was visible in vehicle (VEH) injected mice in either brain area. In unilateral PFF-treated mice (PFF UNI), there was very little or no staining visible in the striatum of the transplanted side (hMG) and only sparse staining in the substantia nigra area. When PFFs were injected to both sides of the striatum (PFF BILAT), but microglia progenitors only transplanted to one side, we observed clear α-synuclein pS129 staining in the non-transplanted side (NT), compared to the transplanted side (hMG) where we observe little to no staining.

However, since our overarching aim was to explore human microglia transplantation as a more relevant model of Parkinson’s disease, we used a paradigm where α-synuclein PFFs were injected to the mice prior to transplantation. In this setup, we injected α-synuclein PFFs/vehicle to the striatum and 30 days later transplanted human microglia progenitors, which remained there for a further 30 days before tissue collection and immunohistochemistry (Fig. 3B). As above, no staining was observed in VEH treated. In both PFF-treated groups, clear α-synuclein pS129 staining was observed in both striatum and substantia nigra. Quantification of the number of α-synuclein pS129 inclusions in the hMG substantia nigra showed significant differences between the paradigms in PFF-treated groups (Fig. 3C). Therefore, we observe that the presence of human microglia prior to injection of PFFs results in reduced spread of the α-synuclein from striatum to substantia nigra, which does not occur with mouse microglia nor when the PFFs are injected before the transplantation of human microglia. One-way ANOVA *F*(5,39) = 17.05, *P* < 0.0001. Tukey’s multiple comparison post hoc testing: PFF UNI → hMG vs. hMG → PFF UNI *P* = <0.0001; PFF BILAT → hMG vs. hMG → PFF BILAT *P* = 0.0003. These differences did not appear to be due to the shorter timepoint of PFF treatment as the number of α-synuclein pS129 positive inclusions in the NT side for the hMG → PFF BILAT group was not significantly different from the NT side of the PFF BILAT → hMG group (Fig. 3D).

### 3.3. Striatal transplantation of human microglia protects tyrosine hydroxylase neurons and reduces small alpha-synuclein aggregates in the substantia nigra

**Fig. 4.**
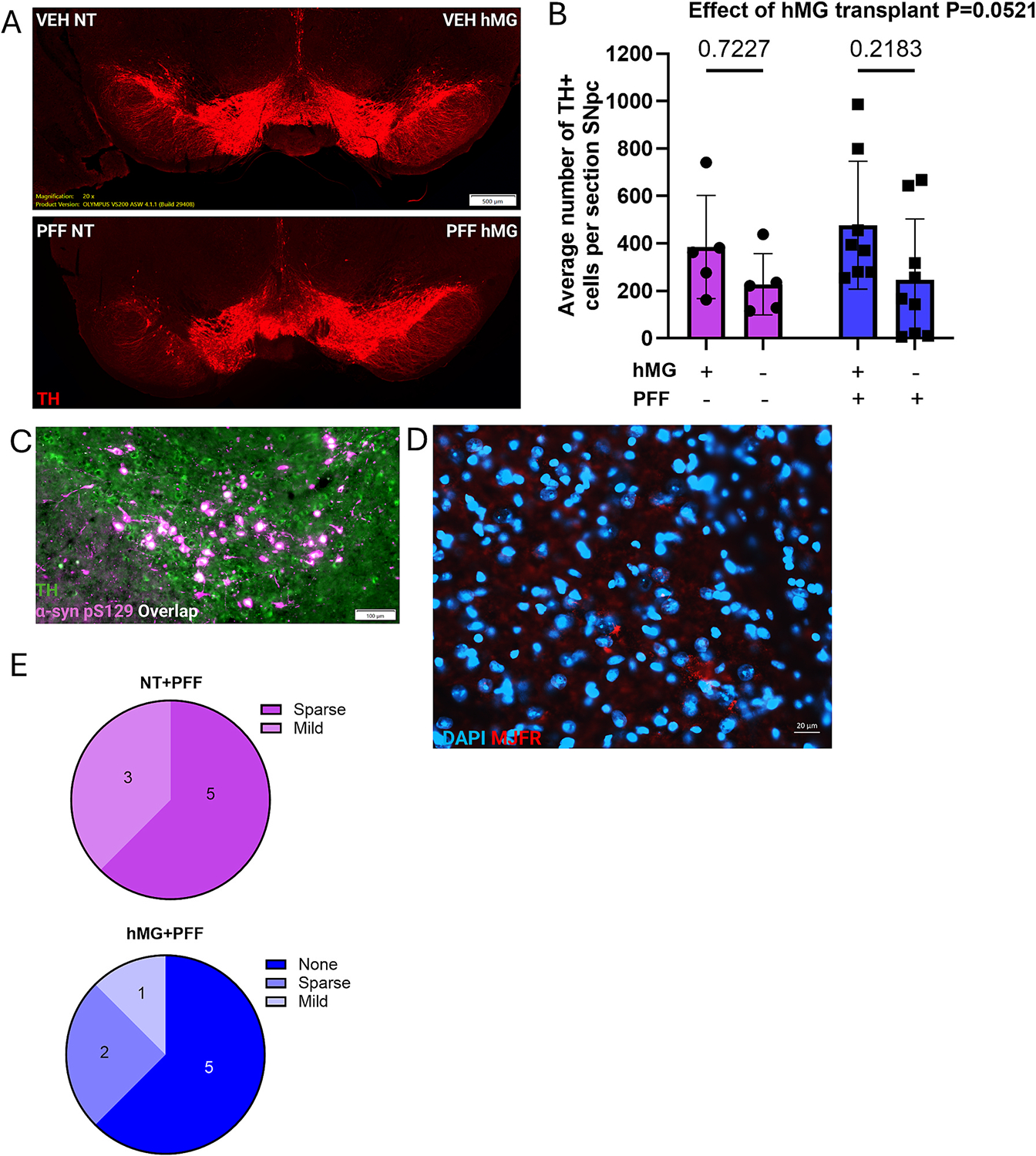
Transplantation of human microglia to striatum is neuroprotective of tyrosine hydroxylase neurons in the substantia nigra pars compacta and reduces small aggregates there. (A) Tyrosine hydroxylase (TH) IHC of substantia nigra from vehicle (VEH)-treated mice and PFF bilateral-treated mice from setup (Fig. 3B). Scale bar 500 μm. (B) Quantification of the number of TH-positive cells in the substantia nigra pars compacta comparing VEH-treated and PFF-treated hMG (transplanted) and NT (non-transplanted) sides. Data represented as the average number of counted cells for 5–6 sections for each mouse. Mean ± SD. (C) Immunohistochemistry of PFF-injected substantia nigra pars compacta for TH (green) and pS129 α-synuclein (magenta), overlap white. Scale bar 100 μm. (D) Image of α-synuclein aggregate antibody [MJFR-14-6-4-2] immunohistochemistry from PFF-injected mouse. Scale bar 20 μm. (H) Pathology scoring counts, i.e. number of mice, for α-synuclein aggregate antibody [MJFR-14-6-4-2] evaluating the hMG and NT side pathology in substantia nigra area of mice injected with PFFs bilaterally.

We next checked whether injection of α-synuclein PFFs would result in tyrosine hydroxylase (TH) cell loss in the substantia nigra pars compacta in the model and whether this could be protected by transplantation of human microglia progenitors. In the paradigm above, where α-synuclein PFFs were injected 30 days before transplantation (Fig. 3B), we observed a clear reduction in TH-positive cell bodies in the substantia nigra pars compacta on the NT side compared to the hMG side in several mice (Fig. 4A). Analysis of the number of TH-positive cells shows a main effect of the human microglia transplantation (two-way ANOVA *F*(1, 23) = 4.197, *P* = 0.0521) (Fig. 4B), indicating that the microglia are neuroprotective of TH+ neurons in this paradigm. To check if the presence of microglia could modify α-synuclein aggregation in this paradigm, we immunostained for pS129 α-synuclein and the α-synuclein aggregate antibody [MJFR-14-6-4-2] in the substantia nigra. While there was clear staining of pS129 α-synuclein in the TH-positive neurons of the substantia nigra pars compacta (Fig. 4CF), we did not observe a significant difference in the PFF BILAT group in total number of inclusions in the hMG vs. NT sides (Fig. 3E). Small aggregate pathology was observed in the substantia nigra area of PFF-injected mice (Fig. 4D) and each side was evaluated using pathology scoring. We observed sparse or mild pathology in all 8 mice evaluated on the NT side (Fig. 4E). In the hMG side, we observed either mild (1/8), sparse (2/8), or no pathology (6/8).

### 3.4. Alpha-synuclein preformed fibrils affect human microglia at the transcriptome level

**Fig. 5.**
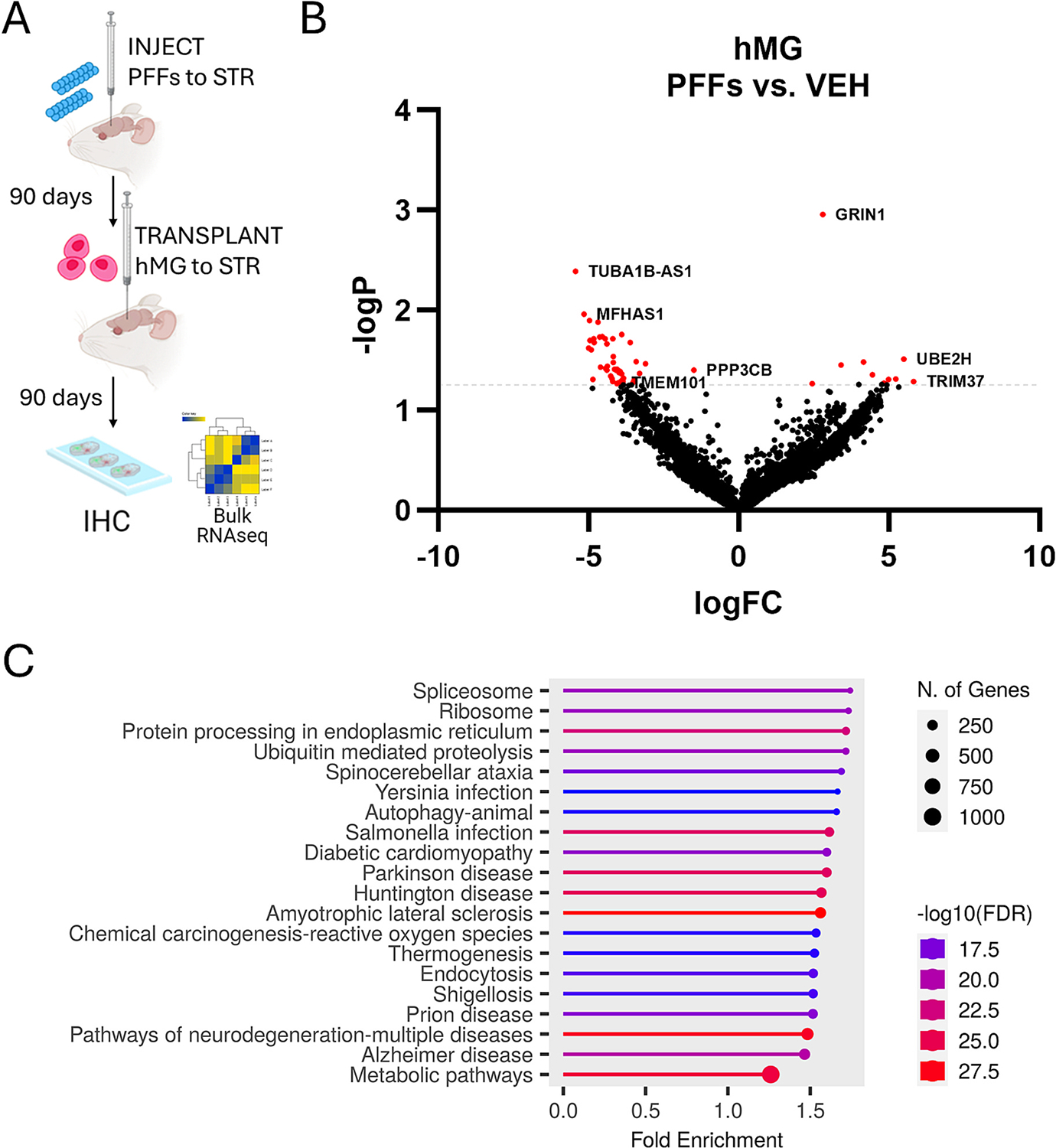
Transplanted human microglia are affected by preformed fibrils. (A) Extended experimental timeline: α-synuclein PFFs were injected to the striatum (STR) 90 days prior to transplantation of human microglia progenitors, and 90 days later, immunohistochemistry (IHC) and bulk RNAseq were performed. (B) Volcano plot of significantly upregulated and downregulated human genes in microglia treated with PFFs compared to vehicle (VEH). Data represented as log fold change (FC) with *P* < 0.05. (C) Pathway analysis of all changed human genes comparing PFFs with VEH.

We further investigated whether extended α-synuclein PFF treatment (90 days before transplant) in concert with a longer transplantation time (90 days) would reveal different outcomes at both the gene and protein levels (total PFF treatment time 120 days) (Fig. 5A). We found 56 human genes were significantly up or downregulated (*P* < 0.05) in transplanted human microglia from the mice treated with PFF vs. vehicle (Fig. 5B). Several of these genes were associated with multiple pathways illustrated in Fig. 5C (Table 2).

Pathways of interest related to PFF treatment of transplanted human microglia from the pathway analysis are for example, protein processing in endoplasmic reticulum, ubiquitin mediated proteolysis, autophagy, Parkinson’s disease, endocytosis, prion disease, pathways of neurodegeneration, and metabolic pathways (Fig. 5C).

### 3.5. Microglia xenotransplantation exerts a neuroprotective effect on mouse hypocretin neurons of the hypothalamus

**Fig. 6.**
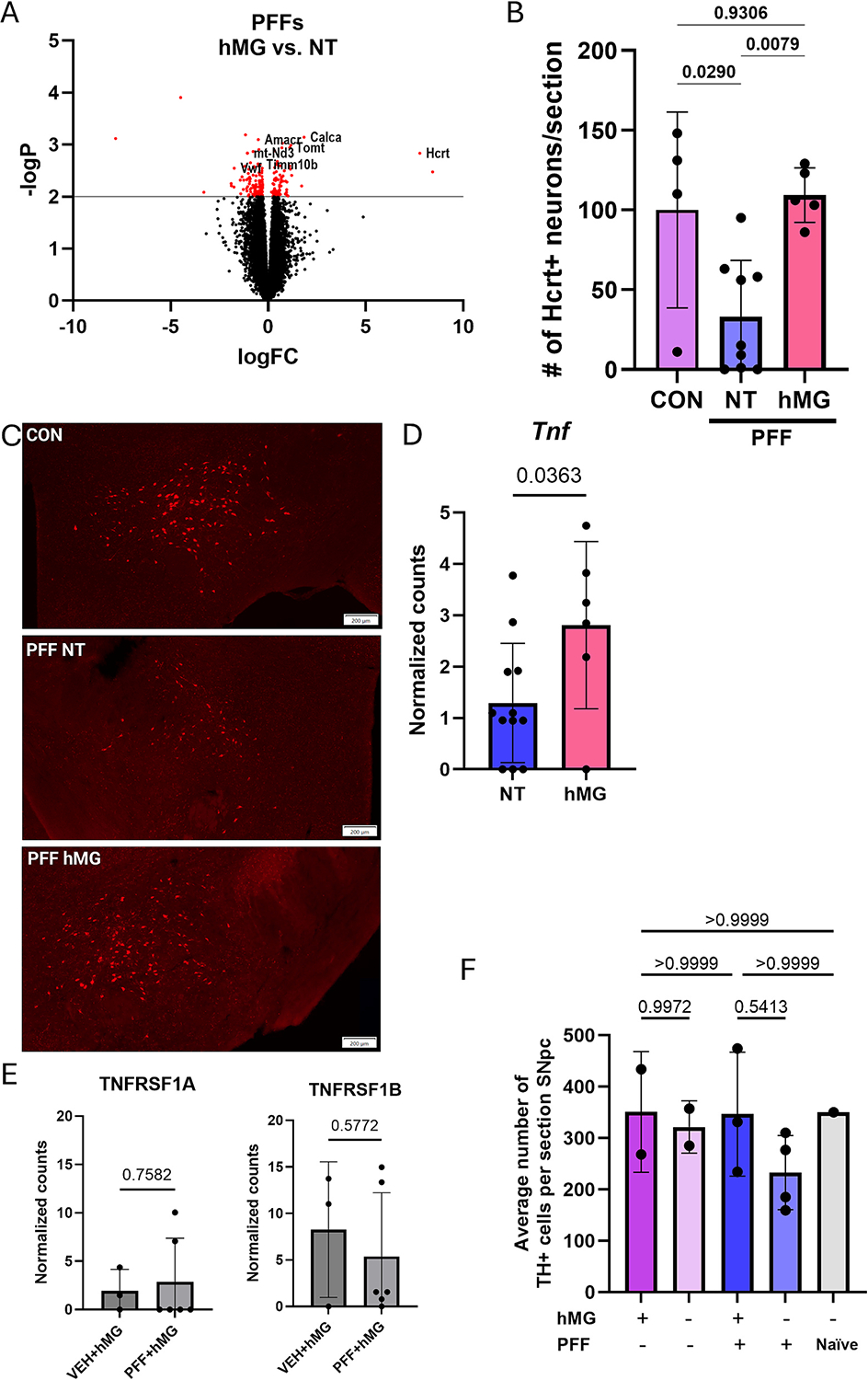
Transplantation of human microglia to the mouse striatum protects hypocretin neurons of the hypothalamus. (A) Volcano plot of significantly upregulated and downregulated mouse genes in mice injected with PFFs and transplanted (hMG) or not (NT). Data as logFC with *P* < 0.01. (B) Quantification of the number of hypocretin-positive (Hcrt+) neurons in the hypothalamus of control (CON, no injections), NT, and hMG PFF-treated mice. Mean ± SD. (C) Representative IHC images of hypocretin staining in the hypothalamus. Scale bar 200 μm. (D) Normalized gene count of mouse *Tnf* from striatum of hMG and NT mice. Mean ± SD. (E) Normalized gene counts of human TNF-α receptors in human microglia transplanted to the mouse brain at 3-months post-transplant comparing vehicle (VEH) and PFF-treated mice. (F) Average number of tyrosine hydroxylase (TH) positive cells in the substantia nigra pars compacta of mice injected with PFFs (120 days) and transplanted with human microglia (90 days).

In the above paradigm (Fig. 5A), we also analysed whether transplantation of human microglia would have any effect on the mice treated with α-synuclein PFFs. A total of 202 mouse genes were significantly up/downregulated (*P* < 0.01) in the mice treated with PFFs comparing hMG vs. NT (Fig. 6A). While the RNAseq analysis revealed several genes of interest, *Hcrt* stood out as having both a fold increase of 7.77 and p-value of 0.0015, therefore we opted to explore this further at the protein level. There are direct and indirect pathways between hypothalamus and striatum (Sakurai et al., 2005), as well as hypocretin receptors present there (Mir et al., 2024). We observed a significant increase in the number of hypocretin-positive neurons in the hypothalamus area on the transplanted (hMG) side compared to the non-transplanted side (NT) (Fig. 6B) (one-way ANOVA *F*(2, 15) = 7.905, *P* = 0.0045; Tukey’s post hoc test for multiple comparisons *P* = 0.0079). Further, the number of hypocretin-positive neurons in the hMG side did not differ significantly from the control side (no injections) (one-way ANOVA, Tukey’s post hoc test for multiple comparisons *P* = 0.9306). Representative images of hypocretin immunostaining (Fig. 6C). Of interest to our study, tumour necrosis factor alpha (TNF-α), a proinflammatory cytokine released by microglia (Gao et al., 2002), has been shown to regulate hypocretin expression, where increased TNF-α expression downregulated hypocretin expression (Zhan et al., 2019). However, our expression data showed a significant increase in expression of mouse TNF-α in hMG striatum (Fig. 6D) (unpaired two-tailed *t*-test *t*(16) = 2.285, *P* = 0.0363). Conversely, no changes were observed in the expression of human microglia TNF-α receptors in PFF compared to vehicle treated mice (Fig. 6E).

We also checked the number of TH-positive cells in the substantia nigra from this cohort of mice. While we observed a similar trend to the shorter timepoint paradigm from the previous experiments, where the microglia were neuroprotective against TH loss in the substantia nigra, the differences were not significant (Fig. 6F).

## 4. Discussion

For the first time, we demonstrate that human microglia progenitors transplanted to the striatum of adult immunodeficient mice survive and express mature microglia markers at up to 3 months post-transplant. Further, the human microglia response to α-synuclein PFFs diverges from mouse response as they prevent propagation of α-synuclein pS129-positive aggregates from striatum to substantia nigra. This does not occur in wild-type mice injected with α-synuclein PFFs alone. Microglia are the main phagocytosing cells of the brain and both mouse and human microglia have been shown to take up human α-synuclein fibrils (Niskanen et al., 2024; Xiong et al., 2021). Therefore, it was somewhat surprising that only the transplanted human microglia had a significant impact on the number of pS129 α-synuclein aggregates in the substantia nigra after striatal injection of PFFs. This gives further evidence of the divergence between mouse and human microglia and why it is important to use human microglia in neurodegenerative disease studies. Conversely, the paradigm where the α-synuclein PFFs were first injected and then one month later the human microglia were transplanted did not demonstrate similar blocking of aggregate transmission. These different results are likely due to the fact that when human microglia are already present, they immediately uptake some of the injected PFFs. In contrast, if the PFFs are injected without human microglia, they have already started to spread to the substantia nigra area. Part of our study involved comparing human and mouse microglia response to α-synuclein spreading therefore we did not implement any microglia depletion protocols; in the future, detailed exploration of the human-specific response of microglia to α-synuclein PFFs and aggregates in the mouse brain will utilize this to increase the number of interactions.

Even though the paradigms differed in their spreading outcomes, when PFFs were injected bilaterally to the striatum and then microglia transplanted unilaterally, we observed fewer small aggregates in the substantia nigra area comparing the transplanted side to non-transplanted side. The aggregates were mainly observed extracellularly and absent in the vehicle-injected mice. Together, our results indicate an effect of human microglia on α-synuclein aggregation that is otherwise not occurring when only mouse microglia are present, thus providing a more human-relevant model to study α-synuclein aggregation using a whole brain environment. Further, we observed a neuroprotective effect of the human microglia on the TH-positive neurons of the substantia nigra of mice injected first with PFFs then transplanted with human microglia. Interestingly, injection of human α-synuclein PFFs to wild-type or α-synuclein-mutant mouse striatum either does not result in TH-positive cell loss in the substantia nigra or only at four months post-injection (Polinski, 2021). Whereas we observe a loss already at two months post-PFF injection in some cases, perhaps this could be due to a vulnerability of the dopamine neurons in these immunodeficient mice.

To further compare the human and mouse microglia response to α-synuclein PFFs, we used a longer timepoint of PFF treatment (120 days total) and longer transplant time (90 days) and studied response using bulk RNAseq from the striatum. This is the first transcriptomic dataset generated from human microglia transplanted to the immunodeficient adult mouse brain and injected with α-synuclein PFFs. We observed clear gene expression changes in human microglia with α-synuclein PFFs. Some significantly upregulated genes are related to, for example, ubiquitination (*PSMD2*, *TRIM37*, *UBE2H*). Interestingly, *GRIN1*, which encodes a critical subunit of NMDA receptors, was most significantly upregulated. NMDA receptors are present on rodent microglia, and their activation drives a proinflammatory response (Raghunatha et al., 2020). NMDA receptor subunits are involved in synaptic plasticity, and in concert with this we see significant upregulation of *CTTNBP2* (regulation of dendritic spines), *PCLO* (part of presynaptic cytoskeletal matrix, establishing synapse active zones and vesicle trafficking), and *ZFYVE27* (appears to promote neurite formation). α-synuclein PFFs may have induced proinflammation which provided a positive feedback loop for upregulation of these genes in human microglia. Significantly downregulated genes include mitochondria-related genes (*NDUFAB1*, *EIF2S1*, *CCDC90B*, *RPUSD4*, *SELENOH*). Our data in *in vitro* human iPSC-derived human microglia treated with α-synuclein PFFs showed a strong induction of iNOS (Niskanen et al., 2024); taken together this could indicate a deficit in mitochondria function from nitric oxide, induced by the PFFs and/or inflammation caused by PFF treatment. Conversely, other significantly downregulated genes were *IL10RB* (active interleukin 10 receptor complex), *ERAP2* (antigen presentation in ER, facilitating epitope presentation for major histocompatibility complex (MHC) class I molecules), *RFXAP* (binds to the X box motif of certain MHC class II gene promoters). Increased proinflammation and upregulation of antigen presentation in microglia are associated with age and neurodegenerative diseases (Fan et al., 2024). While IL-10 is considered an anti-inflammatory cytokine that produces a neuroprotective effect in pathological conditions, both increased and decreased levels have been observed in patients with Parkinson’s disease, indicative of a complex time course of this molecule and its receptors (Porro et al., 2020). Thus, downregulation of antigen presentation genes and anti-inflammatory cytokine receptor in human microglia in response to α-synuclein PFFs could be a protective mechanism at this timepoint.

Since we observed some difference in TH-positive cells between the hMG and NT sides at the shorter PFF 60-day timepoint, we also compared hMG and NT in PFF-treated mice at their gene level. We found significantly changed expression of mouse genes related to mitochondria and ER: upregulation of *Calca* (calcium regulation), *Tomt* (ER-located, positive regulator of protein import), *Nudt13* (involved in NADH processes), and downregulation of *mt-Nd3* (mitochondrial gene, Parkinson’s risk factor), and *Timm10b* (mitochondrial transport). This could indicate a potential protective effect of the human microglia against PFFs in mouse mitochondria and ER but needs further study. Importantly, we found a marked increase in expression of *Hcrt*, the gene encoding hypocretin precursor and giving rise to hypocretin-1 and −2 (also referred to as orexin A and B). Hypocretin is mainly associated with narcolepsy but the pathway is also linked to Parkinson’s disease, likely related to the non-motor symptom of sleep disturbances (Alrouji et al., 2023). Hypocretin neurons in the hypothalamus are reduced in Parkinson’s disease patients and there was evidence of Lewy pathology there, however they did not co-stain, indicating the cells with Lewy bodies were already lost or they are dying via a different mechanism (Thannickal et al., 2007). In our model, we observe loss of hypocretin-positive neurons in the hypothalamus after 120-day α-synuclein PFF treatment, which is protected by transplantation of human microglia. Intriguingly, it was shown that TNF-α can modulate hypocretin expression. Since microglia are known to release TNF-α, we checked expression levels in our model but paradoxically observed a significant increase in expression on the microglia-transplanted side. This is likely due to a transient effect that cannot be observed at the gene or protein level but would need to be followed temporally. Nonetheless, this points to the possibility of an immunomodulatory effect of human microglia in our model. To our knowledge it has not been shown that striatal injection of α-synuclein PFFs to rodent brain leads to hypocretin neuron loss. Since hypocretin deficiency is associated with REM sleep behaviour disorder in narcolepsy (Knudsen et al., 2010), and presence of this symptom has been shown as an early predictor to develop synucleinopathy (Iranzo et al., 2013), including Parkinson’s, this model could be further used for studying prodromal aspects as of the disease and in searching for a disease-modifying therapy.

By demonstrating the survival of the transplanted human microglia in the adult mouse brain and maintenance of a mature phenotype, their ability to reduce α-synuclein aggregation and protect neurons we provide a more disease-relevant model to study these cells *in vivo* to investigate α-synuclein aggregation and downstream neuronal outcomes.

## Figures and Tables

**Table 1:** Primary antibodies used in the studies.

Antibody	Concentration	Producer, catalogue #
Rabbit anti-alpha-synuclein aggregate antibody [MJFR-14-6-4-2]	1:5000	Abcam, ab214033
Mouse anti-human nuclei	1:500	Chemicon, MAB1281
Mouse anti-hypocretin-1 (orexin A)	1:500	R&D Systems, MAB763
Rabbit anti-Iba1	1:1000	Wako, 019–19741
Rabbit anti-P2RY12	1:2000	Sigma-Aldrich, HPA014518
rabbit anti-pS129 alpha-synuclein [EP1546Y]	1:10 000	Abcam, ab51253
rabbit anti-TMEM119	1:1000	Abcam, ab185333
rat anti-TREM2	1:500	Abcam, ab86491
rabbit anti-tyrosine hydroxylase	1:1000	Sigma, AB152

**Table 2:** Significantly changed human genes in PFF-vs. VEH-treated transplanted microglia associated with multiple pathways.

Gene	Up/downregulation	Function
GRIN1	↑	Subunit of NMDA receptors.
EIF2S1	↓	Activator of mitophagy.
NDUFAB1	↓	Enables mitochondrial large ribosomal subunit binding activity.
PPP3CB	↓	Enables ex. calmodulin binding activity; involved in ex. positive regulation of lysosome organization and positive regulation of protein localization to nucleus.
PSMD2	↑	Proteasome 26S Subunit Ubiquitin Receptor, maintenance of protein homeostasis; may be involved in TNF signalling pathway.
TRIM37	↑	May be involved in protein-protein and/or protein-nucleic acid interactions.
NEDD4	↓	Ubiquitin ligase in the ubiquitin proteasome system of protein degradation.
UBE2H	↑	Member of the E2 ubiquitin-conjugating enzyme family.

## Data Availability

The RNAseq data is available publicly.

## References

[R1] Abud EM, Ramirez RN, Martinez ES, , 2017. iPSC-derived human microglia-like cells to study neurological diseases. Neuron 94 (2), 278–293.e9. 10.1016/j.neuron.2017.03.042. 28426964 PMC5482419

[R2] Albert K, Raymundo DP, Panhelainen A, , 2021. Cerebral dopamine neurotrophic factor reduces α-synuclein aggregation and propagation and alleviates behavioural alterations in vivo. Mol. Ther 10.1016/j.ymthe.2021.04.035. PMC8417450 33940158

[R3] Alrouji M, Al-Kuraishy HM, Al-Gareeb AI, Zaafar D, Batiha GES, 2023. Orexin pathway in Parkinson’s disease: a review. Mol. Biol. Rep 50 (7), 6107–6120. 10.1007/s11033-023-08459-5. 37155018

[R4] Bushnell Brian. BBMap. https://sourceforge.net/projects/bbmap/.

[R5] Chatterjee D, Sanchez DS, Quansah E, , 2019. Loss of one engrailed1 allele enhances induced α-synucleinopathy. J. Parkinson’s Disease 9 (2), 315–326. 10.3233/JPD-191590. 30932894 PMC6597991

[R6] Chen Y, Chen L, Lun ATL, Baldoni PL, Smyth GK, 2025. edgeR v4: powerful differential analysis of sequencing data with expanded functionality and improved support for small counts and larger datasets. Nucleic Acids Res. 53 (2), gkaf018. 10.1093/nar/gkaf018. 39844453 PMC11754124

[R7] Fan H, Zhang M, Wen J, , 2024. Microglia in brain aging: an overview of recent basic science and clinical research developments. J. Biomed. Res 38 (2), 122–136. 10.7555/JBR.37.20220220. 38403286 PMC11001587

[R8] Gao HM, Jiang J, Wilson B, Zhang W, Hong JS, Liu B, 2002. Microglial activation-mediated delayed and progressive degeneration of rat nigral dopaminergic neurons: relevance to Parkinson’s disease. J. Neurochem 81 (6), 1285–1297. 10.1046/j.1471-4159.2002.00928.x. 12068076

[R9] Ge SX, Jung D, Yao R, 2020. ShinyGO: a graphical gene-set enrichment tool for animals and plants. Bioinformatics 36 (8), 2628–2629. 10.1093/bioinformatics/btz931. 31882993 PMC7178415

[R10] Gosselin D, Skola D, Coufal NG, , 2017. An environment-dependent transcriptional network specifies human microglia identity. Science 356 (6344). 10.1126/science.aal3222. PMC5858585 28546318

[R11] Hasselmann J, Coburn MA, England W, , 2019. Development of a chimeric model to study and manipulate human microglia in vivo. Neuron 103 (6). 10.1016/j.neuron.2019.07.002, 1016–1033.e10. 31375314 PMC7138101

[R12] Holmqvist S, Lehtonen Š, Chumarina M, , 2016. Creation of a library of induced pluripotent stem cells from Parkinsonian patients. NPJ Parkinsons Dis. 2, 16009. 10.1038/npjparkd.2016.9. 28725696 PMC5516589

[R13] Iranzo A, Tolosa E, Gelpi E, , 2013. Neurodegenerative disease status and post-mortem pathology in idiopathic rapid-eye-movement sleep behaviour disorder: an observational cohort study. Lancet Neurol. 12 (5), 443–453. 10.1016/S1474-4422(13)70056-5. 23562390

[R14] Knudsen S, Gammeltoft S, Jennum PJ, 2010. Rapid eye movement sleep behaviour disorder in patients with narcolepsy is associated with hypocretin-1 deficiency. Brain 133 (2), 568–579. 10.1093/brain/awp320. 20129934

[R15] Koskuvi M, Lehtonen Š, Trontti K, , 2022. Contribution of astrocytes to familial risk and clinical manifestation of schizophrenia. Glia 70 (4), 650–660. 10.1002/glia.24131. 34936134 PMC9306586

[R16] Love MI, Huber W, Anders S, 2014. Moderated estimation of fold change and dispersion for RNA-seq data with DESeq2. Genome Biol. 15 (12), 550. 10.1186/s13059-014-0550-8. 25516281 PMC4302049

[R17] Luk KC, Kehm V, Carroll J, , 2012. Pathological α-synuclein transmission initiates Parkinson-like neurodegeneration in nontransgenic mice. Science 338 (6109), 949–953. 10.1126/science.1227157. 23161999 PMC3552321

[R18] Mahul-Mellier AL, Burtscher J, Maharjan N, , 2020. The process of Lewy body formation, rather than simply α-synuclein fibrillization, is one of the major drivers of neurodegeneration. PNAS 117 (9), 4971–4982. 10.1073/pnas.1913904117. 32075919 PMC7060668

[R19] Mancuso R, Van Den Daele J, Fattorelli N, , 2019. Stem-cell-derived human microglia transplanted in mouse brain to study human disease. Nat. Neurosci 22 (12), 2111–2116. 10.1038/s41593-019-0525-x. 31659342 PMC7616913

[R20] Mancuso R, Fattorelli N, Martinez-Muriana A, , 2024. Xenografted human microglia display diverse transcriptomic states in response to Alzheimer’s disease-related amyloid-β pathology. Nat. Neurosci 1–15. 10.1038/s41593-024-01600-y. PMC11089003 38539015

[R21] McQuade A, Coburn M, Tu CH, Hasselmann J, Davtyan H, Blurton-Jones M, 2018. Development and validation of a simplified method to generate human microglia from pluripotent stem cells. Mol. Neurodegener 13. 10.1186/s13024-018-0297-x. PMC6303871 30577865

[R22] Mir S, Keenan RJ, Bron R, , 2024. The distribution of Hypocretin/Orexin receptor mRNA in the mouse and human brain. Med. Drug Discovery 24, 100202. 10.1016/j.medidd.2024.100202.

[R23] Niskanen J, Peltonen S, Ohtonen S, , 2024. Uptake of alpha-synuclein preformed fibrils is suppressed by inflammation and induces an aberrant phenotype in human microglia. Glia. 10.1002/glia.24626. PMC11660540 39435593

[R24] Patro R, Duggal G, Love MI, Irizarry RA, Kingsford C, 2017. Salmon provides fast and bias-aware quantification of transcript expression. Nat. Methods 14 (4), 417–419. 10.1038/nmeth.4197. 28263959 PMC5600148

[R25] Patterson JR, Kochmanski J, Stoll AC, , 2024. Transcriptomic profiling of early synucleinopathy in rats induced with preformed fibrils. NPJ Parkinsons Dis. 10 (1), 7. 10.1038/s41531-023-00620-y. 38172128 PMC10764951

[R26] Penttinen AM, Suleymanova I, Albert K, Anttila J, Voutilainen MH, Airavaara M, 2016. Characterization of a new low-dose 6-hydroxydopamine model of Parkinson’s disease in rat. J. Neurosci. Res 94 (4), 318–328. 10.1002/jnr.23708. 26762168

[R27] Polinski NK, 2021. A summary of phenotypes observed in the in vivo rodent alpha-synuclein preformed fibril model. JPD 11 (4), 1555–1567. 10.3233/JPD-212847. 34486988 PMC8609716

[R28] Porro C, Cianciulli A, Panaro MA, 2020. The regulatory role of IL-10 in neurodegenerative diseases. Biomolecules 10 (7), 1017. 10.3390/biom10071017. 32659950 PMC7407888

[R29] Raghunatha P, Vosoughi A, Kauppinen TM, Jackson MF, 2020. Microglial NMDA receptors drive pro-inflammatory responses via PARP-1/TRMP2 signaling. Glia 68 (7), 1421–1434. 10.1002/glia.23790. 32036619

[R30] Rathinam C, Poueymirou WT, Rojas J, , 2011. Efficient differentiation and function of human macrophages in humanized CSF-1 mice. Blood 118 (11), 3119–3128. 10.1182/blood-2010-12-326926. 21791433

[R31] Rostami J, Mothes T, Kolahdouzan M, , 2021. Crosstalk between astrocytes and microglia results in increased degradation of α-synuclein and amyloid-β aggregates. J. Neuroinflamm 18 (1), 124. 10.1186/s12974-021-02158-3. PMC8173980 34082772

[R32] Sakurai T, Nagata R, Yamanaka A, , 2005. Input of orexin/hypocretin neurons revealed by a genetically encoded tracer in mice. Neuron 46 (2), 297–308. 10.1016/j.neuron.2005.03.010. 15848807

[R33] Scheiblich H, Dansokho C, Mercan D, , 2021. Microglia jointly degrade fibrillar alpha-synuclein cargo by distribution through tunneling nanotubes. Cell. 10.1016/j.cell.2021.09.007. PMC8527836 34555357

[R34] Scheiblich H, Eikens F, Wischhof L, , 2024. Microglia rescue neurons from aggregate-induced neuronal dysfunction and death through tunneling nanotubes. Neuron. 10.1016/j.neuron.2024.06.029. 39059388

[R35] Smajić S, Prada-Medina CA, Landoulsi Z, , 2022. Single-cell sequencing of human midbrain reveals glial activation and a Parkinson-specific neuronal state. Brain 145 (3), 964–978. 10.1093/brain/awab446. 34919646 PMC9050543

[R36] Tansey MG, Wallings RL, Houser MC, Herrick MK, Keating CE, Joers V, 2022. Inflammation and immune dysfunction in Parkinson disease. Nat. Rev. Immunol 1–17. 10.1038/s41577-022-00684-6. PMC8895080 35246670

[R37] Thannickal TC, Lai YY, Siegel JM, 2007. Hypocretin (orexin) cell loss in Parkinson’s disease. Brain 130 (Pt 6), 1586–1595. 10.1093/brain/awm097. 17491094 PMC8762453

[R38] Trudler D, Nazor KL, Eisele YS, , 2021. Soluble α-synuclein–antibody complexes activate the NLRP3 inflammasome in hiPSC-derived microglia. Proc. Natl. Acad. Sci 118 (15), e2025847118. 10.1073/pnas.2025847118. 33833060 PMC8054017

[R39] Xiong M, Xia D, Yu H, , 2024. Microglia process α-synuclein fibrils and enhance their pathogenicity in a TREM2-dependent manner. Adv. Sci. (Weinh) 12 (7), 2413451. 10.1002/advs.202413451. 39665233 PMC11831461

[R40] Xu R, Li X, Boreland AJ, , 2020. Human iPSC-derived mature microglia retain their identity and functionally integrate in the chimeric mouse brain. Nat. Commun 11 (1), 1577. 10.1038/s41467-020-15411-9. 32221280 PMC7101330

[R41] Yildirim-Balatan C, Fenyi A, Besnault P, , 2024. Parkinson’s disease-derived α-synuclein assemblies combined with chronic-type inflammatory cues promote a neurotoxic microglial phenotype. J. Neuroinflamm 21, 54. 10.1186/s12974-024-03043-5. PMC10882738 38383421

[R42] Zhan S, Che P, Zhao XK, , 2019. Molecular mechanism of tumour necrosis factor alpha regulates hypocretin (orexin) expression, sleep and behaviour. J. Cell Mol. Med 23 (10), 6822–6834. 10.1111/jcmm.14566. 31386303 PMC6787512

